# β-catenin nuclear translocation in colorectal cancer cells is suppressed by PDE10A inhibition, cGMP elevation, and activation of PKG

**DOI:** 10.18632/oncotarget.6705

**Published:** 2015-12-21

**Authors:** Kevin Lee, Ashley S. Lindsey, Nan Li, Bernard Gary, Joel Andrews, Adam B. Keeton, Gary A. Piazza

**Affiliations:** ^1^ Drug Discovery Research Center, Mitchell Cancer Institute, University of South Alabama, Mobile, Alabama, USA; ^2^ Department of Biochemistry and Molecular Genetics, The University of Alabama at Birmingham, Birmingham, Alabama, USA; ^3^ Mitchell Cancer Institute, University of South Alabama, Mobile, Alabama, USA

**Keywords:** β-catenin, PKG, PKA, PDE10A, colon cancer

## Abstract

Phosphodiesterase 10A (PDE10) is a cGMP and cAMP degrading PDE isozyme that is highly expressed in the brain striatum where it appears to play an important role in cognition and psychomotor activity. PDE10 inhibitors are being developed for the treatment of schizophrenia and Huntington's disease and are generally well tolerated, possibly because of low expression levels in most peripheral tissues. We recently reported high levels of PDE10 in colon tumors and that genetic silencing of PDE10 by siRNA or inhibition with small molecule inhibitors can suppress colon tumor cell growth with a high degree of selectivity over normal colonocytes (Li et al., *Oncogene* 2015). These observations suggest PDE10 may have an unrecognized role in tumorigenesis. Here we report that the concentration range by which the highly specific PDE10 inhibitor, Pf-2545920 (MP-10), inhibits colon tumor cell growth parallels the concentration range required to increase cGMP and cAMP levels, and activates PKG and PKA, respectively. Moreover, PDE10 knockdown by shRNA reduces the sensitivity of colon tumor cells to the growth inhibitory activity of Pf-2545920. Pf-2545920 also inhibits the translocation of β-catenin to the nucleus, thereby reducing β-catenin mediated transcription of survivin, resulting in caspase activation and apoptosis. PDE10 mRNA was also found to be elevated in colon tumors compared with normal tissues. These findings suggest that PDE10 can be targeted for cancer therapy or prevention whereby inhibition results in cGMP elevation and PKG activation to reduce β-catenin-mediated transcription of survival proteins leading to the selective apoptosis of cancer cells.

## INTRODUCTION

Phosphodiesterase 10 (PDE10) was first identified in 1999 by three groups independently [1]–[3]. PDE10 is a dual substrate PDE that can hydrolyze both cyclic guanosine monophosphoate (cGMP) and cyclic adenosine monophosphate (cAMP). Unlike other PDE isozymes, PDE10 is expressed at high levels in the brain striatum but has limited expression in peripheral tissues in humans [4]. In the central nervous system PDE10 is known to mediate the responsiveness of striatal medium spiny neurons to dopaminergic transmission as shown by studies using PDE10A knockout mice [5]. There are two known isoforms of PDE10A in humans, PDE10A1 which is cytosolic, while PDE10A2 contains 10 extra amino acids at the N-terminal domain, consisting of a membrane binding domain and a protein kinase A (PKA) phosphorylation motif [6]. The majority of research being conducted on PDE10 is in the field of neurobiology where there is evidence that PDE10 inhibitors are effective in animal models of schizophrenia [7], [8] and in Huntington's disease [9].

Data from our lab has shown that colon tumor cells have high PDE10 mRNA and protein levels compared to normal colonocytes [10]. We also showed that silencing PDE10 through siRNA/shRNA, or by pharmacological inhibition, suppresses the growth of colon tumor cells with minimal effects on normal colonocytes. PDE10 inhibitors also induce caspase activation, reduce proliferation, and induce cell cycle arrest in colon tumor cells. PDE10 knockdown studies confirmed these results, as well as linking the suppression of β-catenin signaling with the activation of protein kinase G (PKG) resulting from PDE10 inhibition and the elevation of intracellular cGMP levels. PKG has also been associated to the synthesis of cell cycle regulating proteins [11], and to β-catenin signaling in cancer cells [10], [12]–[16]. To date, there have been no reports in the literature, besides our recent publication, implicating PDE10A in tumorigenesis with no reports suggesting that PDE10 inhibitors could be used to treat malignant diseases [10]. Here we show that the highly selective PDE10 inhibitor, Pf-2545920, can suppress colon tumor cell growth through cGMP elevation and the activation of PKG. As evidence of its selectivity, knockdown of PDE10 by shRNA reduced the sensitivity of the cells to Pf-2545920 treatment. PDE10 inhibition led to an increase in cGMP and cAMP which in turn activated the downstream kinases PKG and PKA, respectively, as shown through phosphorylation of the PKG substrate VASP. Through the use of pharmacological inhibitors of PKG and PKA it was determined that cGMP elevation was critical for the growth inhibitory activity of Pf-2545920. PDE10 inhibition also led to a reduction in the levels of β-catenin in the cytoplasm and nucleus resulting in decreased survivin expression, leading to an increase in caspase cleavage and the induction of apoptosis. We also show that PDE10 mRNA was increased in colon tumors compared with peripheral tissues from mice.

## RESULTS

### PDE10 inhibitor, Pf-2545920, inhibits colon tumor cell growth and induces apoptosis

**Figure 1:**
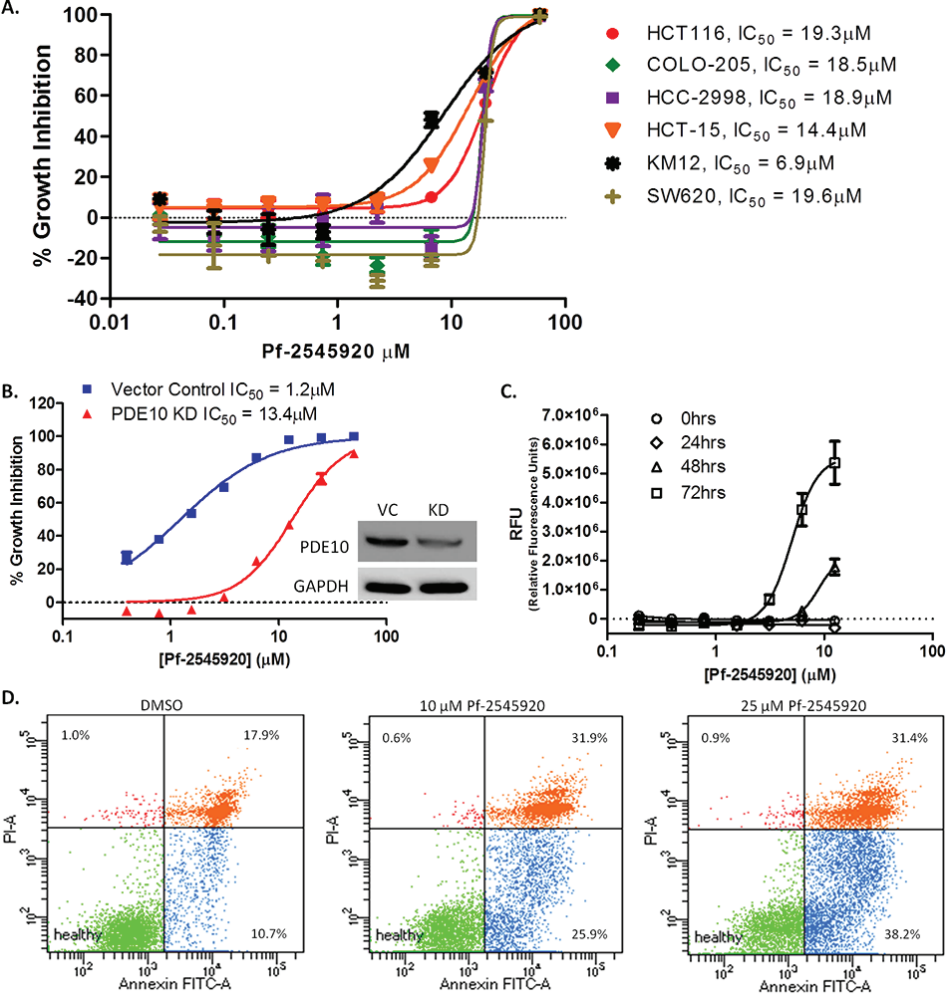
PDE10 as a target for colon cancer **A.** A panel of human colon tumor cell lines were tested for growth inhibitory activity of PDE10 inhibitor Pf-2545920 in dose response for 72 hours. **B.** HT-29 PDE10 knockdown cells were tested for growth inhibitory activity of Pf-2545920 against vector control cells. **C.** Cytotoxicity assay was performed in the presence of Pf-2545920 in dose response over time and measured as fluorescence intensity. **D.** HT-29 cells were incubated with DMSO, 10 μM Pf-2545920, or 25 μM Pf-2545920 for 72 hours before staining with Annexin V and analyzed by FACS.

Human colon cancer cell lines were previously reported to have high levels of PDE10 compared with normal colonocytes and that treatment using selective PDE10 inhibitors can inhibit colon tumor cell growth. The highly selective PDE10 inhibitor, Pf-2545920, was studied for growth inhibitory activity in a panel of nine human colon tumor cell lines. Isozyme selectivity of Pf-2545920 is shown in Table 1. As shown in Figure 1A, Pf-2545920 comparably inhibited the growth of all lines with IC_50_ values ranging from 6.9 μM to 19.6 μM. To confirm that the growth inhibitory activity of Pf-2545920 is mediated by PDE10 inhibition, PDE10 levels were suppressed by shRNA. As shown in Figure 1B, shRNA PDE10 knockdown reduced sensitivity of HT-29 colon tumor cells to growth inhibition when treated with Pf-2545920 from an IC_50_ of 1.2 μM in vector control cells to an IC_50_ of 13.4 μM in PDE10 knockdown cells. A fluorescent cytotoxicity assay was used to further characterize the tumor cell growth inhibitory activity of Pf-2545920. As shown in Figure 1C, Pf-2545920 induces cell death in a concentration-dependent manner at concentrations that parallel its growth inhibitory activity. The possibility that the mechanism of cell death involved apoptosis was determined by measuring Annexin V levels. As shown in Figure 1D, Pf-2545920 induced apoptosis in a dose-dependent manner as evidenced by an increased percentage of cells showing positive staining for Annexin V, as a marker of apoptosis, and/or propidium iodide (PI), a marker of cell death. Treatment with vehicle control shows 10.7% of cells staining positive for Annexin V and 17.9% for Annexin V and PI. Treatment with 10 μM Pf-2545920 increased the percentage of cells undergoing apoptosis to 25.9% showing positive staining for Annexin V and 31.9% for both Annexin V and PI. Treatment with 25 μM Pf-2545920 further increased the percentage of cells undergoing apoptosis with 38.2% showing positive staining for Annexin V and 31.4 % for both Annexin V and PI.

**Table 1:** Phosphodiesterase specificity of Pf-2545920 is presented for multiple PDE isoforms for both cGMP and cAMP

Isozyme	cGMP IC_50_	cAMP IC_50_
PDE1A	Inactive @ 10 μM	
PDE2A	>50 μM	36.6 μM
PDE3A	>50 μM	>50 μM
PDE3B	Inactive @ 10 μM	
PDE4B2	N/A	2.36 μM
PDE5	8.6 μM	N/A
PDE9A	Inactive @ 10 μM	N/A
PDE10A	0.156 nM	0.588 nM
PDE11A	Inactive @ 10 μM	

**Figure 2:**
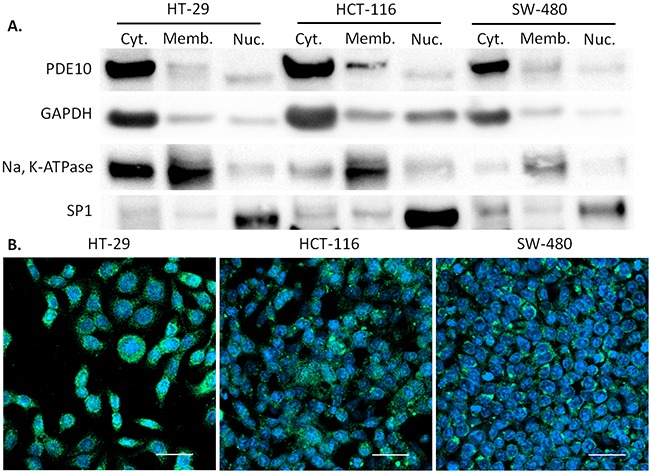
Subcellular localization of PDE10 in human colon tumor cell lines **A.** Subcellular fractions of colon tumor cell lines HT-29, HCT-116, and SW-480 were analyzed for expression of PDE10. GAPDH served as loading control for cytoplasmic fraction. Na, K-ATPase served as loading control for membrane fraction. SP1 served as loading control for nuclear fraction. **B.** Confocal immunofluorescence microscopy of PDE10 with secondary antibody conjugated to Alexa Fluor 488 merged with DAPI for HT-29, HCT-116, and SW-480. Scale bar = 50μm.

To confirm that colon tumor cells express PDE10 and to determine the relative expression of PDE10A1, a cytoplasmic enzyme, and PDE10A2, a membrane bound enzyme, subcellular localization studies were performed. The majority of PDE10 in three colon tumor cell lines, HT-29, SW-480, and HCT-116 was found to be distributed in the cytoplasm as shown by Western blot in Figure 2A, and by confocal immunofluorescence microscopy in Figure 2B.

### Inhibition of PDE10 by Pf-2545920 activates downstream kinases as shown by VASP phosphorylation in colon tumor cells

**Figure 3:**
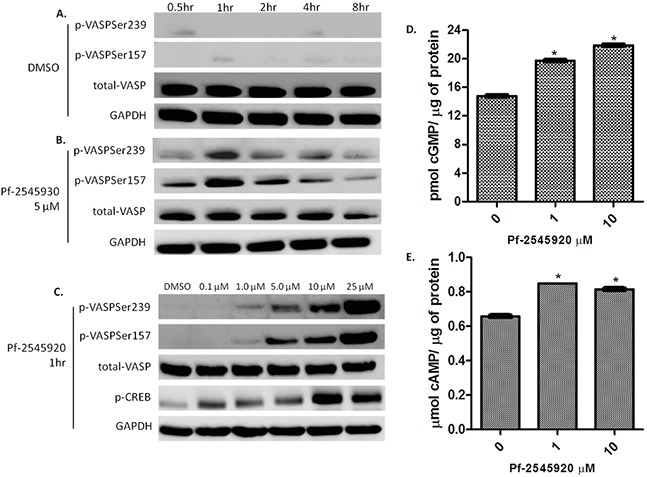
PDE10 inhibitors induce downstream kinase signaling **A.** VASP phosphorylation at serine 239, PKG site, and serine 157, PKA site, in HCT-116 colon tumor cells treated with vehicle control DMSO over a time course of 8 hours. **B.** Pf-2545920 treatment at 5μM induces VASP phosphorylation at serine 239 as well as serine 157 in a time-dependent manner. **C.** HCT-116 cells treated in dose response with Pf-2545920 for 1 hour induces phosphorylation of VASP at serine 239 and at serine 157 in a concentration-dependent manner. Pf-2545920 treatment induces an increase in **D.** cGMP, and **E.** cAMP in a concentration-dependent manner. Statistical significance was assayed using a Student's T-test. *P<0.0001.

PKG activation has been previously reported by numerous groups to suppress colon tumor cell growth, while PKA activation does not appear to be involved [10], [12], [17]–[19]. To determine the signaling mechanisms resulting from PDE10 inhibition, vasodilator-stimulated protein (VASP) was used as a marker for protein kinase activation in response to treatment. PKG phosphorylates VASP preferentially at serine 239, while PKA phosphorylates VASP preferentially at serine 157 by increasing intracellular levels of cGMP and cAMP, respectively [20], [21]. As a control, treatment of HCT-116 colon tumor cells with vehicle (DMSO) over a time period of 8 hours had no effect on the phosphorylation of VASP, as shown in Figure 3A. Treatment of HCT-116 cells with Pf-2545920 at a concentration of 5 μM increased the level of phosphorylated VASP using phosphospecific antibodies specific for VASP (serine 239) and VASP (serine 157) in a time-dependent manner, as shown in Figure 3B. HCT-116 cells treated with Pf-2545920 for 1 hour activated both PKG and PKA as evidenced by the phosphorylation of VASP, as well as by the phosphorylation of CREB, a known PKA substrate, as shown in Figure 3C [22], [23]. Consistent with these observations, Pf-2545920 treatment of HCT-116 increased intracellular cGMP and cAMP levels, Figure 3D and 3E respectively. The concentration at which Pf-2545920 increases intracellular cGMP and cAMP levels not only parallel the concentration range required to activate PKG and PKA, but also parallels the IC_50_ value to inhibit colon tumor cell growth as shown in Figure 1A.

### Inhibition of PKG, but not PKA, blunts sensitivity of colon tumor cells to growth inhibition by PDE10 inhibitors

**Figure 4:**
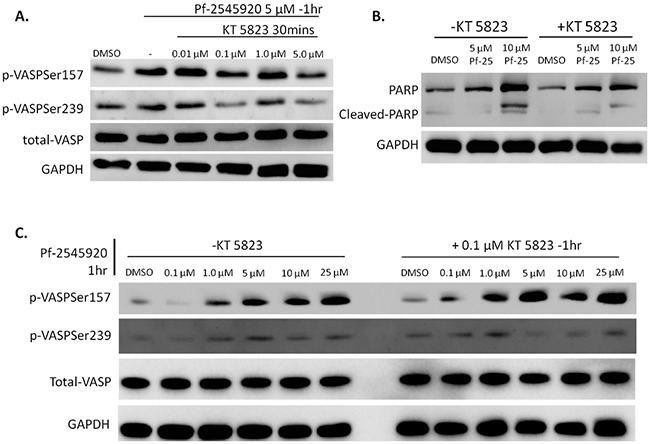
Addition of KT 5823 blocks activity of PKG when stimulated with Pf-2545920 **A.** HT-29 cells were pretreated with KT 5823 for 30 minutes in ascending concentrations before the addition of Pf-2545920 at 5μM for 1 hour to stimulate kinase activity. **B.** HT-29 cells were treated with or without 0.1μM KT 5823 for 30 minutes before the addition of Pf-2545920 at the concentrations indicated for 6 hours and assayed for PARP cleavage. **C.** HT-29 cells were pretreated with or without 0.1μM KT 5823 for 30 minutes before the addition of Pf-2545920 in dose-response for 1 hour to stimulate kinase activity and assayed for VASP phosphorylation.

To discern potential differences that cGMP/PKG and cAMP/PKA signaling may have on colon tumor cell growth, experiments were designed to block downstream kinase activation resulting from PDE10 inhibition. The PKG inhibitor, KT 5823, was used as a probe to block activation of PKG in response to treatment with Pf-2545920. KT 5823 has been previously reported to inhibit the effects from PKG activation in colon tumor cells [24], [25]. HT-29 cells were pretreated for 30 minutes with KT 5823 in ascending concentrations before the addition of 5 μM of Pf-2545920 to activate kinases PKG and PKA, as shown in Figure 4A. Treatment of cells with KT 5823 caused a decrease in PKG activation when analyzed for VASP phosphorylation at serine 239, while minimally affecting the activity of PKA, as evidenced by VASP phosphorylation at serine 157. In order to assess downstream effects of PKG inhibition, HT-29 cells were pretreated with KT 5823 for 30 minutes before the addition of Pf-2545920 at 5 μM and 10 μM for 6 hours. As shown in Figure 4B, the addition of 0.1 μM KT 5823 reduced apoptosis as shown by Western blot for cleaved-PARP. HT-29 cells were pretreated with or without 0.1 μM of KT 5823 for 30 minutes before the addition of Pf-2545920. As shown in Figure 4C, PKG activity is decreased in response to KT 5823 treatment when compared to control, while the activity of PKA is unchanged.

**Figure 5:**
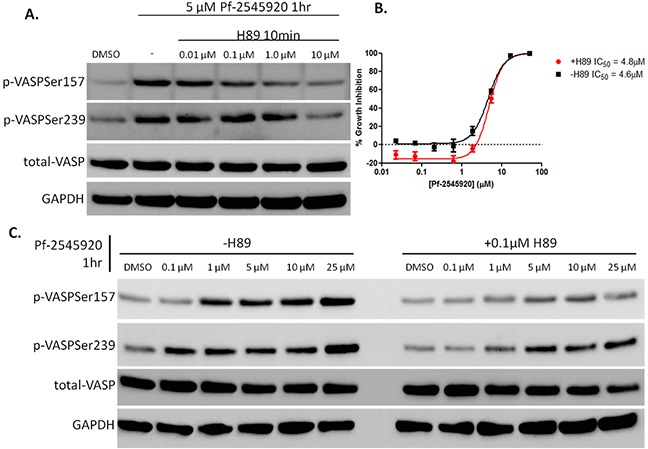
Pharmacological inhibition of PKA using H89 **A.** HT-29 cells were pretreated with H89 in ascending concentrations for 10 minutes before the addition of 5μM of Pf-2545920 to stimulate kinase activity for 1 hour. **B.** HT-29 cells were pretreated with 0.1μM H89 for 10 minutes before the addition of Pf-2545920 in dose-response and incubated for 72 hours at 37 degrees before being assayed for cell growth inhibition by the addition of CellTiter-Glo. **C.** HT-29 cells were pretreated with or without H89 for 10 minutes at 0.1μM before the addition of Pf-2545920 in dose-response for 1 hour to stimulate PKA activity and assayed for VASP phosphorylation via western blot.

cAMP/PKA signaling was probed using H89, an inhibitor of PKA [26]–[28]. HT-29 colon tumor cells were pretreated with H89 for 10 minutes before being treated with Pf-2545920 to stimulate kinase signaling. As shown in Figure 5A, pretreatment with H89 blocks the activation of PKA in a concentration-dependent manner, while also blocking the activation of PKG, albeit at higher concentrations. This is consistent with reports that H89 has the ability to inhibit other kinases at higher doses [26]. A low dose of H89 was chosen to selectively inhibit PKA activity, while having minimal effects on the activity of PKG. HT-29 cells that were pretreated with H89 for 10 minutes prior to the addition of Pf-2545920 for 72 hours had no effect on the sensitivity of the cells to the growth inhibitory activity of Pf-2545920, as shown in Figure 5B. However, pretreatment of HT-29 cells with H89 before the addition of Pf-2545920 for 1 hour greatly reduced the activity of PKA as evidenced by the lack of phosphorylation of VASP at serine 157, while minimally affecting PKG phosphorylation of VASP at serine 239 as shown in Figure 5C.

### Treatment of tumor cells with Pf-2545920 induces apoptosis through a β-catenin mediated pathway

**Figure 6:**
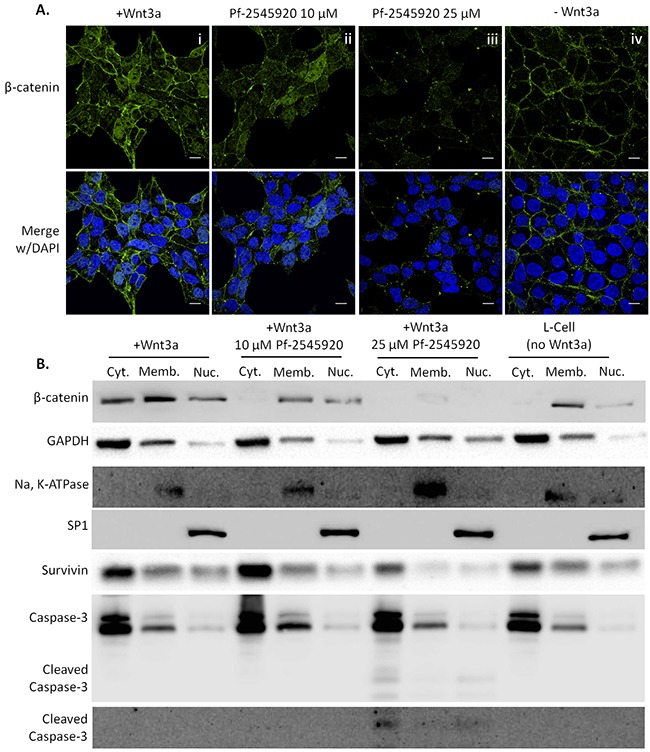
PDE10 inhibition using Pf-2545920 inhibits β-catenin translocation to the nucleus in SW-480 colon tumor cells Representative confocal immunofluorescent images of β-catenin are shown in **A.** i) β-catenin translocation was stimulated by the addition of Wnt3a conditioned media for 5 hours. ii) Pretreatment of cells with 10μM Pf-2545920 for 30 minutes reduces the amount of β-catenin in the nucleus upon Wnt3a stimulation. iii) Pretreatment of cells with 25μM Pf-2545920 for 30 minutes further reduces the amount of β-catenin able to translocate to the nucleus in addition to reducing total amounts of β-catenin protein levels. iv) Localization of β-catenin is found predominantly at the membrane under basal conditions without Wnt3a stimulation. **B.** Subcellular fractionation of SW-480 cells under the same conditions as A were assayed by western blot for β-catenin as well as its transcriptional product survivin. Lysates were also probed for the activation of caspase cleavage with two distinct antibodies. Cell fractionation was confirmed by analyzing fractionation controls: GAPDH for cytoplasm; Na, K-ATPase for membrane; and SP1 for nuclear. Cyt.= cytoplasmic fraction, Memb.= membrane fraction, Nuc.= nuclear fraction. Scale bar = 10μm.

Human SW-480 colon tumor cells were used to determine the translocation of β-catenin into the nucleus where it binds to the Tcf/Lef transcription factor and induces the transcription of survival proteins such as survivin and cyclin D1 [10], [16]–[18], [29]. Translocation into the nucleus was stimulated using conditioned media from Wnt3a secreting cells. If Wnt ligand is not present, β-catenin not bound to cadherins junctions at the membrane is bound by the APC/Axin/GSK complex where it is phosphorylated and ubiquitinated by β-TRCP before being degraded by the proteasome. Wnt ligand binds to the Frizzled receptor on the cell surface which results in intracellular phosphorylation of the Dishevelled protein. Upon phosphorylation, Dishevelled binds to Axin resulting in dissociation of the APC/Axin/GSK complex, thereby allowing β-catenin to bypass the destruction complex and enter the nucleus [29]. As shown in Figure 6A by confocal immunofluorescence microscopy, pre-incubation of SW-480 colon tumor cells with 10 μM Pf-2545920 for 30 minutes reduced the amount of β-catenin in the nucleus (Panel ii) compared to control (Panel i), while total protein levels of β-catenin are decreased when treated with 25 μM Pf-2545920 (Panel iii). Basal levels of β-catenin in the membrane are shown (Panel iv) without Wnt stimulation. Similar results were seen by Western blot following subcellular fractionation, Figure 6B. With no Wnt stimulation the majority of β-catenin was found in the membrane fraction. Upon stimulation with Wnt, β-catenin was found to be localized in the membrane fraction, the cytoplasmic fraction, and the nuclear fraction. Treatment with 10 μM Pf-2545920 reduced the levels of β-catenin found in the cytoplasmic and nuclear fractions, and treatment with 25 μM Pf-2545920 reduced the total amounts of β-catenin found in the cells. The decrease in β-catenin in the nuclear fraction of treated cells resulted in a decrease in survivin, and an increase in caspase cleavage leading to apoptosis.

### PDE10 expression in normal tissues and colon tumors

**Figure 7:**
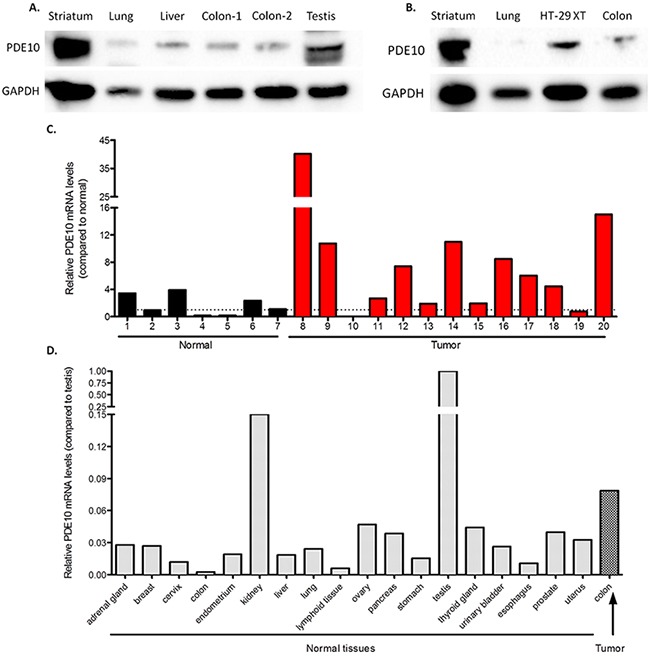
PDE10 expression in normal tissues and colon tumors **A.** Tissues were collected from C57/BL6 mice and analyzed for expression of PDE10. **B.** Tissues were collected from athymic mice and analyzed for expression of PDE10. HT-29XT, tumors established from HT-29 colon tumor cells in athymic mice. **C.** Human tissue cDNA array analyzing colonic tumors versus normal colon. **D.** Human tissue cDNA array analyzing PDE10 mRNA levels from normal tissues normalized to testis.

PDE10 mRNA and protein has been shown to be expressed *in vivo* at low levels in normal tissues in both humans as well as mice, however no reports indicate the expression of PDE10 in malignant tissues [4], [30]. To study PDE10 expression levels further in the context of our previous report showing PDE10 overexpression in colorectal tumors compared with normal colonic mucosa, tissues were collected from C57/BL6 mice and analyzed for the expression of PDE10. Consistent with previous reports of mRNA levels by other investigators, PDE10 protein expression was high in the striatum and in the testis, with a low basal expression observed in the lung, liver, and colon mucosa, Figure 7A [30]. Tissues were also collected from athymic mice and analyzed for PDE10 expression, as shown in Figure 7B. Consistent with results obtained from C57/BL6 mice, PDE10 expression was high in the striatum, with low levels in the lung and the colon mucosa. Colon tumors derived from HT-29 colon tumor cells established in athymic mice (HT-29XT) showed high levels of PDE10 expression in comparison to normal mouse colonic mucosa.

Human PDE10 mRNA levels were also measured in a large number of tissues using cDNA arrays and compared with levels in colon tumors (Origene). As shown in Figure 7C, the majority of colon tumor samples exhibit high PDE10 mRNA when compared to normal colon. In comparison to high levels of PDE10 mRNA levels in testis, all other tissues expressed appreciably lower levels of PDE10 mRNA, as shown in Figure 7D. The average PDE10 mRNA level from colon tumors was higher than all other normal tissues in the array, including a more than 30-fold increase in PDE10 mRNA when comparing to normal colon, with the exception of the kidneys.

## DISCUSSION

PDE10 has mostly been studied in the field of neurobiology where it appears to play an important role in the CNS to regulate motor function and cognition [31]. Stemming from this research, multiple pharmaceutical companies have developed PDE10 inhibitors in which several are in clinical trials for Huntington's disease and schizophrenia. Studies previously published by our group have shown that a PDE10 inhibitor originally designed for use in CNS disorders, Pf-2545920, was able to inhibit colon tumor growth *in vitro*. We also reported that colon tumor cells express high levels of PDE10 in comparison to normal colon epithelial cells and genetic knockdown can selectively suppress growth of colon tumor cells with lesser effects on normal colon epithelial cells [10]. Furthermore, the neurological field has been focused mainly on the regulatory role of PDE10 on cAMP/PKA signaling, where an increase in cAMP activates PKA to mediate gene transcription through the phosphorylation of CREB [31]. Here we show that a clinically relevant PDE10 inhibitor, Pf-2545920, can activate signaling through PKG as well as PKA. The downstream mechanism, as we hypothesized, involves the activation of caspases and induction of apoptosis through the inhibition of β-catenin mediated transcription. The mechanism also involves reduced translocation of β-catenin to the nucleus.

In summary, we showed PDE10 to be localized primarily to the cytoplasm of multiple colon tumor cell lines by Western blots and by immunofluorescence microscopy. Pf-2545920 was shown to inhibit the growth of a panel of human colon tumor cell lines with similar potencies. The PDE10 involvement was confirmed by shRNA knockdown of PDE10 to attenuate the ability of Pf-2545920 to inhibit the growth of colon tumor cells. Pf-2545920 induced cytotoxicity in HT-29 colon tumor cells through the induction of apoptosis, as shown by Annexin V staining levels.

Treatment of human colon tumor cells with Pf-2545920 activated both PKG and PKA, which occurred in a time and concentration-dependent manner, as shown by the phosphorylation of the PKG and PKA substrate VASP, as well as by the PKA substrate CREB. VASP is phosphorylated at serine 157 by PKA and at serine 239 by PKG, while CREB is phosphorylated by PKA at serine 133. The activation of PKG and PKA signaling was a direct result of the increase in cyclic nucleotides when treating colon tumor cells with Pf-2545920 in a concentration-dependent manner.

To determine the importance of PKG and PKA signaling in colon tumor cells treated with PDE10 inhibitors, kinase activity was inhibited prior to the addition of Pf-2545920. Through the use of the small molecule PKA inhibitor, H89, PKA was found to be inhibited at a concentration of 0.1 μM in HT-29 colon tumor cells without significantly affecting PKG activity. Pretreating cells with the PKG inhibitor, KT 5823 at 0.1 μM, inhibited PKG signaling while having minimal effects on PKA activity. It was also shown that pretreating cells with KT 5823 before the addition of Pf-2545920 reduced PARP cleavage by blocking the ability of PKG to induce cell death. The ability of KT 5823 to block PKG signaling was confirmed using Pf-2545920. It should be noted that KT 5823 has been shown to act independently of PKG inhibition thereby producing off-target effects [34]. However, through the use of dose-finding experiments it was determined that 0.1 μM was specific to inhibit PKG signaling but not PKA signaling. Pretreating cells with H89 before the addition of Pf-2545920 showed no effect on tumor cell growth inhibition following 72 hours of treatment, even though PKA signaling was inhibited when treating with Pf-2545920. It should be noted that there is a slight increase seen in phosphorylation of VASP at serine 157 and a slight reduction of phosphorylation at serine 239. These observations suggest that although PKG preferentially phosphorylates VASP at serine 239 and PKA preferentially phosphorylates VASP at serine 157, each kinase does not exclusively phosphorylate its preferred site and can phosphorylate the other serine: serine 157 for PKG and serine 239 for PKA [32], [33].

PKG activation has been reported to inhibit β-catenin mediated transcription [10], [16]–[18]. By employing SW-480 colon tumor cells it was shown that under Wnt3a stimulation, β-catenin translocates from the membrane to the cytoplasm and the nucleus where it can mediate the transcription of survival proteins. This was shown by confocal immunofluorescence microscopy whereby the addition of Pf-2545920 inhibited the translocation of β-catenin to the nucleus at 10 μM, and at a higher concentration of 25 μM reduced the amount of total β-catenin. This data was confirmed by subcellular fractionation followed by western blot. Controls showed that translocation of β-catenin to the nucleus was stimulated with the addition of Wnt3a, and that this translocation was inhibited by Pf-2545920. This was followed by the reduction of survivin, and the activation of caspase-3.

In studies to determine the *in vivo* relevance of PDE10 in colon cancer, PDE10 mRNA levels were found to be increased in colon tumors relative to normal colon as well as in comparison to various other normal tissues from a human tissue cDNA array. At the protein level it was shown that various peripheral tissues obtained from wild type C57/BL6 mice, as well as athymic nude mice, also have low PDE10 protein levels when comparing to the striatum as well as the testis. Meanwhile, xenograft tumors derived from HT-29 colon tumor cells expressed high levels of PDE10 when compared to normal colon mucosa. While there is a low basal expression of PDE10 in various normal peripheral tissues, our results indicate that colon tumors have an over-expression of PDE10 at both the mRNA and at the protein level.

Selectivity of Pf-2545920 indicated that this compound is a highly specific PDE10 inhibitor with IC_50_ values in the subnanomolar range as previously described [35]. With the exception of PDE5 and PDE4, Pf-2545920 showed no inhibitory activity in the range by which tumor cell growth is inhibited. Studies by our lab have indicated that dual inhibition of PDE5 and PDE10 yielded additive effects in tumor cell growth inhibition [36]. In addition treatment with rolipram, a PDE4 inhibitor, did not affect colon tumor cell growth at concentrations up to 100 μM, data not shown. This is consistent with data presented here as well as elsewhere indicating that increasing cAMP to activate PKA in colon tumor cells does not significantly contribute to cell death [19].

**Figure 8:**
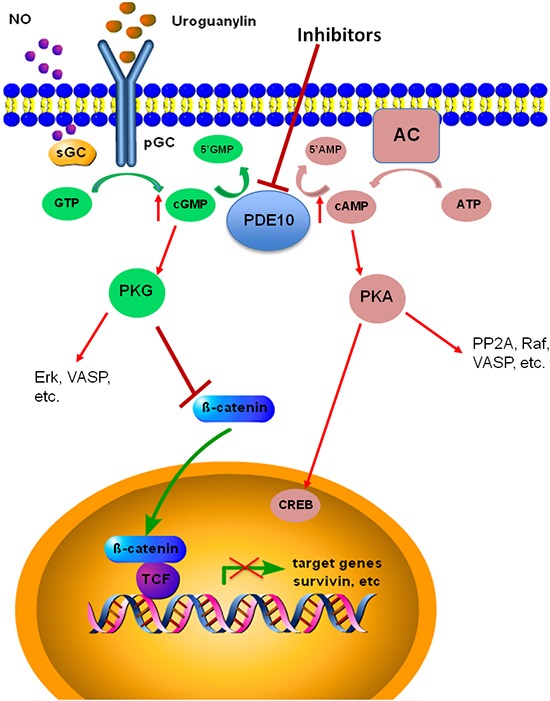
PDE10 signaling in colon cancer The inhibition of PDE10 increases intracellular concentrations of cGMP and cAMP leading to the activation of PKG and PKA, respectively. In colon cancer cells PKG acts to inhibit the nuclear translocation of β-catenin in order to modulate its oncogenic signaling.

In summary, colon tumor cell growth is suppressed by PDE10 inhibition which resulted in an activation of PKG signaling using the selective inhibitor Pf-2545920. Pf-2545920 increased intracellular cGMP levels, leading to the activation of PKG. Translocation of β-catenin to the nucleus was inhibited by PDE10 inhibition thereby reducing β-catenin mediated transcription of survival proteins, as depicted in Figure 8. It was also shown that PDE10 was increased in colon tumors when compared to various normal tissues at the mRNA level from a human tissue cDNA array and at the protein level in C57/BL6 and athymic nude mice tissues analyzed by Western blot. Taken together these observations support further research to determine the role of PDE10 in tumorigenesis and the possibility of using inhibitors for the treatment or prevention of cancer.

## MATERIALS AND METHODS

### Cell culture

Human tumor cell lines were obtained from ATCC and grown in RPMI 1640 medium containing 5% fetal bovine serum.

### Growth inhibition assays

Growth Assays were performed by seeding 5,000 cells per well in a 96-well microtiter plate, or 2,500 cells per well in half-area 96-well microtiter plates. Cells were allowed to settle onto the bottom of the plate before being dosed with compounds in dose-response for 72 hours at 37°C in a humidified incubator containing 5% CO_2._ After incubation, growth inhibitory activity was assayed using the Cell Titer Glo Assay (Promega) according to manufacturer's recommendations.

### cGMP EIA assay

Total levels of cGMP were measured using a competitive ELISA assay (Cayman Chemical) according to the manufacturer's recommendations. A standard curve is generated using samples of known cGMP concentrations provided with the assay and plotted using linear regression to extrapolate samples.

### β-catenin nuclear translocation

Cover glass bottom 35mm tissue culture dishes were precoated with EmbryoMax 0.1% gelatin from Millipore according to manufacturer's recommendations. Cells were then plated and allowed to grow to 70-90% confluency before treatments. SW-480 cells were serum starved overnight prior to the addition of PDE10 inhibitors at the concentrations indicated. Cells were allowed to incubate at 37°C for 1 hour before the addition of conditioned media. Briefly, Wnt3a or parental L-cells lacking Wnt3a were purchased from the ATCC and grown according to the manufacurer's recommendations. Once cells reached confluency, the media was collected and filtered before use. Conditioned media was added to SW-480 cells in order to stimulate nuclear translocation of β-catenin for 5 hours before fixation and staining.

### β-catenin immunofluorescence

Cover glass bottom 35mm tissue culture dishes were precoated with EmbryoMax 0.1% gelatin from Millipore according to manufacturer's recommendations. Cells were then plated and allowed to grow to 70-90% confluency before treatments. Cells were fixed with 10% buffered formalin, before permeabilization with PBS containing 0.1% Trition X-100. Cells were incubated with the appropriate primary antibodies before incubation with secondary antibodies conjugated with Alexa-fluor 488 and mounted using ProLong Gold antifade reagent with DAPI (Molecular Probes-ThermoFisher Scientific). Images were acquired using a Nikon A1 Laser Scanning Confocal microscope. Z-stacks of samples were acquired for analysis of whole cell β-catenin distribution. Images presented are of a single slice through the cells and are representative of the entire sample.

### Western blot

Cell lysates were separated by SDS-PAGE using 12% polyacrylamide gel, 7.5% for PDE10 protein analysis, followed by electrophoretic transfer to nitrocellulose membranes. The membranes were then blocked at room temperature on an orbital shaker for 1 hour in a solution of 5% non-fat dry milk in TBS with 0.1% Tween 20 (TBS-T). Membranes were then incubated overnight at 4 degrees on an orbital shaker with appropriate primary antibodies. Membranes were then washed three times with TBS-T before addition of appropriate secondary antibodies, and incubated at room temperature for 1 hour on an orbital shaker. Membranes were washed and developed using SuperSignal West Enhanced Chemiluminescence Reagent (Pierce).

### Cytotoxicity assay

Cells were plated in 96-well microtiter plates at a denstiy of 5,000 cells per well and treated with compound or vehicle control at the doses and times indicated. Cytotoxicity was measured using CellTox Green Cytotoxicity Assay (Promega) according to the manufacturer's recommendations.

### Apoptosis

Apoptosis was assayed using the Annexin V-FITC + PI apoptosis detection kit from Leinco Technologies according to the manufacturer's recommendations. HT-29 cells were incubated with compounds for 72 hrs at 37°C before being analyzed for apoptosis by FACS.

### Stable knockdown of PDE10

HT-29 PDE10 knockdown cells were generated and described previously [10]. Briefly, lentiviral particles targeting PDE10 were produced in HEK293T cells according to the manufacturer's instruction. HT-29 cells were transduced by the lentivirus particles followed by puromycin selection (5μg/ml) for 2 weeks. Individual cell colonies stably expressing shRNA were selected and isolated in the presence of puromycin, and evaluated by western blotting previously. PDE10 knockdown cells and vector control cells were plated in 96-well micro-titer plates and assayed for growth inhibition to Pf-2545920.
